# Decoding the Flavor Structure of Jiang-Flavor Low-Alcohol Base Baijiu: A Machine Learning-Driven Approach to Reveal the Flavor Evolution Patterns and Key Quality Control Nodes

**DOI:** 10.3390/foods15111891

**Published:** 2026-05-27

**Authors:** Jiaxing Geng, Yongguang Huang

**Affiliations:** 1 College of Liquor and Food Engineering, Guizhou University, Guiyang 550025, China; 18275004719@163.com; 2 Key Laboratory of Fermentation Engineering and Biological Pharmacy of Guizhou Province, College of Liquor and Food Engineering, Guizhou University, Guiyang 550025, China

**Keywords:** low-alcohol base baijiu, volatile flavor compounds, SHAP analysis, flavor markers, classification model

## Abstract

Low-alcohol base baijiu, a by-product of Jiang-flavor baijiu production, contains abundant flavor compounds, yet its flavor structure remains insufficiently understood, limiting its high-value utilization. This study integrated flavoromics with interpretable machine learning to characterize the flavor structure of Jiang-flavor low-alcohol base baijiu and identify its key driving factors. During distillation, ethanol decreased from 38.67% to 6.50% (*v*/*v*), whereas total acidity increased from 2.74 to 7.53 g/L and pH decreased from 3.01 to 2.59, accompanied by a sensory transition from harmonious and full-bodied notes to sour and salty attributes. Based on sensory and flavor component analysis, the samples can be divided into three gradients according to ethanol volume fraction (>20%, 10–20%, <10%), and their flavor profiles exhibit a systematic evolution: as alcohol content decreases, the relative content of esters and alcohols decreases, while the relative content of acids increases. Random Forest identified 3-methyl-1-butanol, furfuryl ethyl ether, and tetramethyl pyrazine as key markers positively associated with harmonious and smooth attributes (r > 0.93) and negatively associated with sour and salty notes. Odor activity value analysis further quantified stage-dependent sensory contributions. The XGBoost model accurately predicted flavor stages (accuracy = 0.955), and SHAP analysis highlighted low-threshold esters and acids as critical regulators of flavor quality. These findings provide a quantitative and interpretable framework for precision blending, quality grading, and the valorization of low-alcohol base baijiu.

## 1. Introduction

In recent years, the global spirits market has kept growing and is expected to reach about USD 656.5 billion by 2025, and baijiu is a major part of this growth. In China, baijiu is still the main spirit category, and it shows a pattern of lower output but steady quality. In 2024, large-scale enterprises produced 4.145 million kiloliters of baijiu, with a sales revenue of CNY 796.384 billion and profits of CNY 250.865 billion. Jiang-flavor baijiu is known for its high quality and high price. As interest in low-alcohol products increases, low-alcohol base baijiu has become important. In particular, low-alcohol fractions from Jiang-flavor baijiu still have strong flavor features and a high potential for use [1,2]. These fractions also contain key chemical information that reflects the fermentation and process changes [3]. However, their composition, flavor structure, and value have not been studied in a complete and systematic way yet.

Traditional flavoromics relies on high-throughput analytical platforms, such as GC–MS and GC × GC–MS, to generate large-scale flavor datasets and uses chemometric methods to establish multilevel associations between chemical composition and sensory attributes for the identification, characterization, and prediction of key aroma compounds [4,5]. For example, in Jiang-flavor baijiu, the integration of GC–MS, sensory evaluation, and odor activity value (OAV) analysis enabled the identification of 201 major volatile compounds and the screening of 37 signature markers that discriminate among different base-baijiu types, demonstrating the utility of this workflow for baijiu typing [6]. Nevertheless, traditional approaches may exhibit amplified discrepancies between instrumental quantification, sensory perception, and limited extrapolation robustness when confronted with high dimensionality, multicollinearity, matrix effects, and the inherent subjectivity of sensory evaluation [7]. Moreover, the physiological state and subjective experience of sensory panelists can further weaken the reproducibility of the results [8,9]. To break these limitations, machine learning has demonstrated significant advantages in food flavor analysis and quality evaluation by virtue of its ability to automatically learn patterns from data and make predictions [10]. For example, reducing the dependence on artificial thresholds and subjective experience has been systematically verified in related fields [11]. Additionally, through deep learning and other models, the complex mapping between production process parameters and flavor characteristics can be effectively fitted, which can ensure prediction accuracy while improving model generalization ability [12]. Moreover, the comprehensive use of F1-score, Matthews correlation coefficient (MCC), and area under the receiver operating characteristic curve (AUC–ROC) and other multiple metrics, can provide a more comprehensive and robust performance evaluation in the multiclassification task with category imbalance, avoiding the bias caused by a single accuracy metric [13]. This evaluation system has developed a consensus in food and wine quality modeling research.

In this study, we systematically characterized Jiang-flavor low-alcohol base baijiu throughout distillation by tracking dynamic changes in ethanol content, pH, total acidity, and other physicochemical parameters. These measurements were integrated with sensory evaluation and quantitative flavor-component data to elucidate flavor structure evolution and differences between rounds. Key difference compounds were then identified using a Random Forest model, and their sensory contributions were assessed via odor activity values (OAVs). Building on these features, multiple machine learning models were developed and compared to enable the accurate prediction of base-baijiu quality grades. Finally, Shapley Additive Explanations (SHAP) were applied to interpret the direction and mechanisms of influence of the most important compounds.

## 2. Materials and Methods

### 2.1. Materials and Reagents

The samples were obtained from different rounds of distilled low-alcohol base baijiu with different levels of spirits brewed in 2024 by the Moutai brewery in Guizhou. The first three samples were taken at 1 min intervals, and the subsequent samples were taken at 2 min intervals up to 39 min, totaling 756 samples. The sensory and physical–chemical characteristics and some samples came from the upper, middle, and lower levels of each round and from the mixed samples of the four shifts, with 21 samples in each round, numbered 1–21 sequentially, totaling 63 samples in the three rounds. According to GB/T 20821-2007 [14] “Baijiu Produced by the Liquid Method,” the alcohol content range for low-alcohol base spirits is 0% vol to 40% vol.

### 2.2. Main Instruments and Equipment

FULI-Chromatec Crystal9000 Gas Chromatograph-Mass Spectrometer; quartz capillary column DB-WAX, 30 m × 0.25 mm × 0.25 μm (Agilent, Santa Clara, CA, USA); portable densitometer DMA35 (antonpaar, Graz, Austria); SPME Fiber Assembly (Supelco, Bellefonte, PA, USA); automatic potentiometric titrator; pure water/ultrapure water all-in-one machine (Think-lab, Göttingen, Germany); Mettler pH meter s220 (Mettler Inc., Columbus, OH, USA); standard wine glass; and so on.

### 2.3. Sensory Description Analysis

Based on GB/T 33405-2016 [15] and a modified literature method [16], a comprehensive sensory evaluation of baijiu was conducted covering color, aroma, taste, and overall character, with flavor profiles constructed for key aroma and taste attributes. The sensory evaluation panel consisted of nine members, including two national judges, four provincial judges, and three graduate students; the average age was 34. The evaluation employed a blind tasting method, with three rounds of repeated tastings conducted on a total of 63 samples to systematically extract the sensory attributes of each sample. By collecting and compiling sensory descriptors and classifying them according to the alcoholic beverage flavor classification system, a flavor wheel for low-alcohol base baijiu was constructed. Based on this flavor wheel, the nine most frequently occurring sensory descriptors (sorghum aroma, jiang-aroma, ethanol aroma, watery taste, pickled vegetable flavor, floral aroma, sour taste, salty taste, bitter taste, smooth, and harmonious) were selected as characteristic sensory indicators to distinguish between different low-alcohol base baijiu samples. A 9-point rating scale (0–9 points with 0.5-point intervals) was used for scoring, and statistical calculations were performed on the scores of each sample. All sensory evaluations in this study complied with ethical guidelines: participants were provided with a detailed explanation of the experiment prior to tasting and gave written informed consent; all data were processed and analyzed anonymously to protect participant privacy.

### 2.4. Measurement of Physical and Chemical Indicators

Alcohol content was measured using a portable densitometer Anton Paar DMA 35, the pH of the baijiu was determined by a Mettler pH meter s220 (Mettler Inc., USA), and the total acid was determined according to the National Standard for Food Safety—Determination of Total Acid in Food (GB12456-2021) [17]. All the samples involved in the experiments were tested three times in parallel.

### 2.5. Analysis of Volatile Flavor Compounds

**Sample treatment:** The method was further optimized according to the literature [18]. Specifically, ultrapure water was used to dilute the samples to 10% (*v*/*v*), and the samples with less than 10% (*v*/*v*) did not need to be diluted. 10 mL of diluted or undiluted samples were accurately measured and placed into a 20 mL headspace injection bottle, saturated with 3 g of NaCl, and then 20 μL of mixed internal standards (2-octanol, ethyl 2-ethylbutyrate, and amyl acetate) were added, and then vortexed with the cap tightly closed to mix.

**HS-SPME method:** Using SPME fiber head, the sample was preheated at 50 °C for 10 min, extracted and adsorbed for 40 min, and then injected into the sample, and then desorbed at 250 °C for 5 min. The test operation was repeated three times under the same conditions.

**GC conditions:** DB-WAX column (30 m × 0.25 mm × 0.25 μm, Agilent Technologies) was used, the temperature of the injection port and the GC detector was 250 °C, helium was used as the carrier gas (purity ≥99.999%), and the flow rate was 1 mL/min, with no shunt injection. The programmed temperature increase conditions were as follows: initial temperature of 40 °C was held for 2 min. The program heating conditions were as follows: initial temperature of 40 °C held for 2 min, 3 °C/min to 110 °C without holding, 3.5 °C/min to 250 °C without holding.

**MS conditions:** The EI ion source and an electron energy of 70 eV were used, the temperature of the ion source was set to 230 °C, the temperature of the transmission line was set to 230 °C, and the scanning range was *m*/*z* 30~550.

**Qualitative-Quantitative Analysis:** Qualitative analysis was conducted via mass spectrometry with NIST20 library matching, requiring both a mass spectral match factor ≥85% and a retention index (RI) deviation <2% from reference standards for confident identification. Quantitative analysis followed an optimized internal standard method [19], using standard curves (Table S1) based on peak area and concentration ratios to the internal standard. Where standard curves were unavailable, semi-quantification was applied based on the internal standard.

### 2.6. OAV Calculation

The OAV is calculated as the ratio of the actual concentration of the flavor compound in the sample to the olfactory threshold of that compound. The olfactory and gustatory thresholds for flavor compounds were obtained from the literature [20,21,22,23,24,25]. Volatile flavor compounds with an OAV > 1 are considered to be compounds that contribute prominently to overall sensory flavor [26].

### 2.7. Predictive Modeling of Flavor Structure Segmentation

Eight models including MLP Classifier, Decision Tree Classifier, Logistic Regression, Random Forest Classifier, XGB Classifier, K Neighbors Classifier, Gradient Boosting Classifier, and SVC, were selected for predictive model construction for the flavor structure classification of low-alcohol base baijiu. The dataset was randomly divided into separate training and test sets, where 80% of the data were used for training and 20% for testing [27]. Model performance was evaluated using four metrics: accuracy, F1-score, recall, and precision, with the values of the five evaluated metrics usually ranging from 0 to 1. The closer the value is to 1, the better the model performance is [28,29]. The classification performance of the model was also evaluated using the ROC curve area and confusion matrix visualization. The SHAP method in interpretable machine learning can be used for global interpretation [30,31]. This method improves the credibility and transparency of machine learning models by calculating the Shapley value of each feature in the model to rank the feature importance [32]. All machine learning models were completed using Anaconda3 2025.06.0 (Python 3.13.5 64-bit) and Microsoft Visual Studio Code (1.105.1).

### 2.8. Data Processing and Analysis

The data were organized using Excel 2019; Spearman’s correlation analysis was performed using SPSS Statistics 27, and Z-score-standardized hierarchical cluster analysis was conducted; basic graphs and heatmaps were created using Origin Pro 2025, GraphPad Prism 10.1.2, and Adobe Illustrator 2025; machine learning analysis was performed using R (in a Visual Studio Code 1.105.1 environment), with the dataset split into training and testing sets in an 8:2 ratio.

## 3. Results and Analysis

### 3.1. Analysis of the Dynamic Changes in Physicochemical Characteristics of Low-Alcohol Base Baijiu

**Figure 1:**
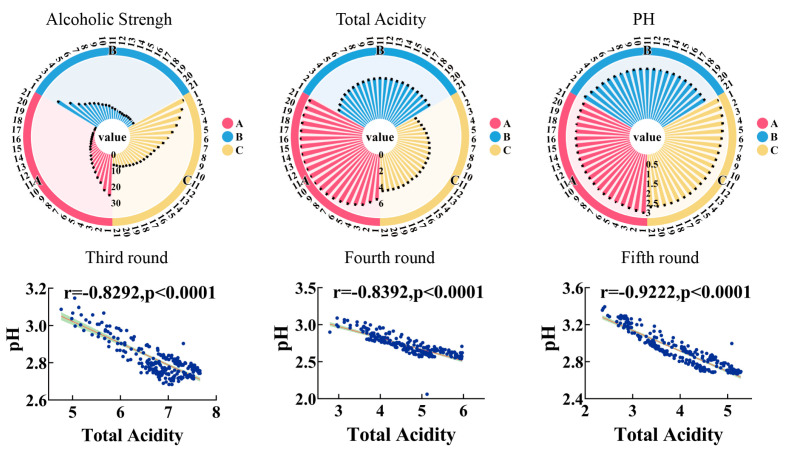
Dynamic changes in physicochemical indexes of low-alcohol base baijiu (A: Third round; B: Fourth round; C: Fifth round; sample size: n = 21).

Physicochemical characteristics are fundamental for evaluating baijiu quality and are closely linked to sensory attributes and flavor composition. Industry standards designate alcohol content, total acidity, and pH as key quality requirements [1], and studies confirm their significant correlation with volatile compounds and sensory perception, supporting their use in process monitoring and quality discrimination [33,34]. Therefore, we selected the batches (the third through fifth) that best represent the typical soy sauce aroma profile, exhibit the finest body, and demonstrate the highest production volume and greatest production stability. We systematically analyzed the dynamic changes in alcohol content, pH, and total acidity of different low-alcohol base liquors, as well as their interactions, to further reveal the evolving trends in the correlations among these physicochemical indicators (Figure 1).

The ethanol concentration of all rounds of low-alcohol base baijiu showed a rapid and then slow gradient-decreasing trend with the advancement of the distillation process (Figure 1), from 38.67% to 6.50%. At the same time, acidity increased (Figure 1). The pH decreased from 3.01 to 2.59, and total acidity rose from 2.74 to 7.53 g/L. These trends indicate an inverse relationship between ethanol concentration and acid accumulation during distillation. Correlation analyses (Figure 1) further showed a highly significant negative correlation between pH and total acidity (*p* < 0.0001), confirming their strong coupling throughout the distillation process.

The above phenomenon is mainly attributed to the differences in volatility and boiling points of the components. Ethanol and some low molecular esters are preferentially distilled out in the pre-distillation stage due to their lower boiling points, resulting in a particularly significant drop in alcohol at the initial stage, and a decrease in the saturation degree of various flavor compounds in the baijiu [35]. In contrast, higher-boiling and more hydrophilic organic acids (e.g., lactic and acetic acids) are more readily entrained as distillation proceeds, and progressively accumulate in low-alcohol base baijiu, resulting in increased total acidity and decreased pH [2]. It is worth noting that core process parameters such as humidity control, temperature control, and fermentation time varied systematically from round to round, which directly led to the round-specificity of the initial values and the magnitude of the changes in alcohol and total acid, reflecting the dominant shaping of metabolites by process design [36,37,38]. These distillation-driven physicochemical gradients provide the material basis for the subsequent evolution of sensory qualities and flavor. However, a deeper scientific question is whether the evolution of sensory qualities is simply linearly related to the decrease in alcohol content? Or are there certain critical nodes driven by key components that lead to fundamental changes in flavor structure? To answer this question, we further analyzed the sensory profile quantitatively over time.

### 3.2. Analysis of the Dynamic Changes in Sensory Characteristics of Low-Alcohol Base Baijiu

**Figure 2:**
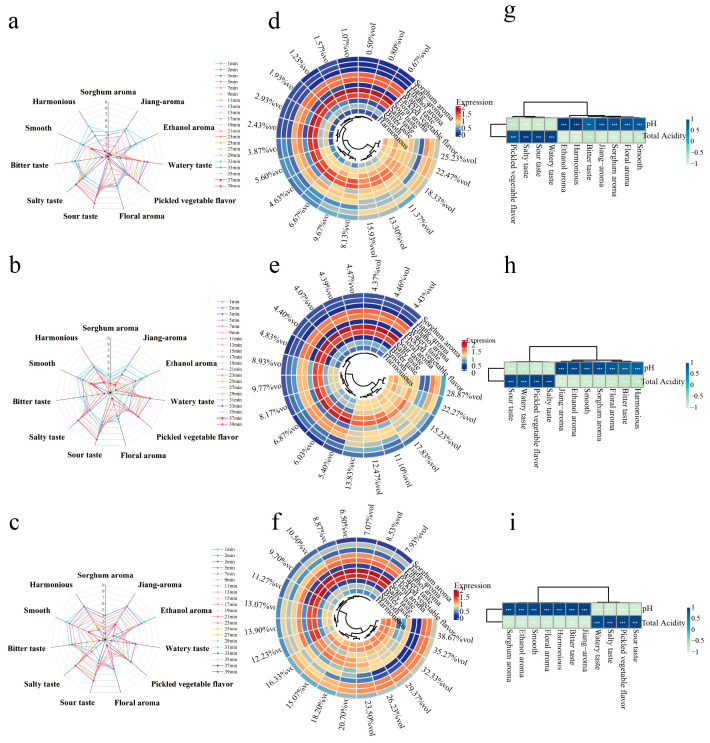
Radar charts showing the dynamic changes in the sensory profile of low-alcohol base baijiu: (**a**) third round; (**b**) fourth round; (**c**) fifth round. Cluster heatmaps showing the dynamic changes in the sensory profile of low-alcohol base baijiu: (**d**) third round; (**e**) fourth round; (**f**) fifth round. Spearman’s correlation analysis of physicochemical parameters and sensory attributes of low-alcohol base baijiu (**: *p* < 0.01; ***: *p* < 0.001): (**g**) third round; (**h**) fourth round; (**i**) fifth round.

Sensory evaluation is an important method for judging baijiu quality and for guiding its use [39]. Temporal sensory analysis across distillation rounds (1–39 min) showed that as distillation progressed, the intensity of positive qualities (sorghum aroma, jiang-aroma, floral aroma, ethanol aroma) decreased, while negative flavors (sour taste, salty taste, watery taste, pickled vegetable flavor) increased, and harmonious and smooth attributes declined accordingly (Figure 2). Quantitative data confirmed this trend: floral aroma decreased from 5.50 to 0.5 in the third round, while sour taste rose from 5.09 to 8.18; jiang-aroma decreased from 5.70 to 1.52 in the fourth round, with sour taste increasing from 5.13 to 8.12; and sorghum aroma dropped from 3.93 to 0.82 in the fifth round, accompanied by a rise in sour taste from 3.10 to 7.05 (Figure 2a–c). The overall flavor structure evolved from aroma harmonization in the high-ethanol early stage to sour–salty dominance later. Notably, the fifth round exhibited a richer, more coordinated initial profile, with higher scores in sorghum aroma (3.93), jiang-aroma (6.50), floral aroma (5.73), and harmonious and smooth (all >6.20). In contrast, sour and salty tastes increased more sharply in the third and fourth rounds, reaching higher intensities (up to 8.12) than in the fifth round (up to 7.13), likely due to the progressive accumulation of acids and salts (e.g., NaCl, KCl, etc.) across rounds [40].

In order to systematically reveal the deep structure of sensory evolution, we performed a clustered heat map analysis of the samples based on alcoholic strength (Figure 2d–f). The analysis results showed that all rounds of low-alcohol base baijiu could be clearly classified into three stages with different sensory characteristics based on the alcohol content. Samples with alcohol content > 20% (*v*/*v*) clustered into Stage I and were characterized by relatively high intensities of positive aromas (e.g., sorghum aroma, jiang-aroma, and floral aroma) together with superior harmonious and smooth attributes. Samples with alcohol content of 10–20% (*v*/*v*) formed Stage II, in which positive attributes declined markedly, sour and salty tastes became more pronounced, and overall balance progressively weakened. Samples with alcohol content < 10% (*v*/*v*) clustered into Stage III, dominated by sourness, saltiness, and pickled vegetable flavor; in this stage, aroma intensity was minimal and overall harmony was substantially diminished. This three-stage flavor structure model based on an alcohol gradient is an important finding of this study. It reveals for the first time, in a quantitative manner, that the evolution of sensory quality is not a linear decline, but that there are two key quality turning points (20% *v*/*v* and 10% *v*/*v*). This phase transition implies that a systematic reorganization of the composition or interactions of flavor substances may have occurred at specific abv thresholds.

To test this hypothesis, we further analyzed the correlation between physicochemical indicators and sensory attributes (Figure 2g–i). Total acidity correlated positively with salty taste, sour taste, watery taste, and pickled vegetable flavor (*p* < 0.001), and negatively with floral aroma, ethanol aroma, jiang-aroma, and harmoniousness (*p* < 0.001). Thus, rising acidity not only enhanced sour–salty perceptions but also weakened the core aroma and overall harmonious and smooth attributes. Conversely, higher pH (lower acidity) corresponded to stronger aroma, better harmoniousness, and smoother mouthfeel. This pattern may reflect the early evaporation of ethanol and short-chain esters, reducing aroma intensity later [35,41]; while the enrichment of higher-boiling organic acids (e.g., lactic, acetic acids) heightened sour–salty notes and impaired mouthfeel [42,43].

Sensory flavor differences exist in low-alcohol base baijiu, which stem from differences in the content and composition of key volatile compounds. In order to reveal the chemical drivers, we further analyzed the quantitative and relative composition of volatile flavor compounds in low-alcohol base baijiu based on the sensory flavor hierarchy.

### 3.3. Analysis of Volatile Flavor Compounds in Low-Alcohol Base Baijiu

#### 3.3.1. Analysis of the Dynamics of Volatile Flavor Compounds in Low-Alcohol Base Baijiu

**Figure 3:**
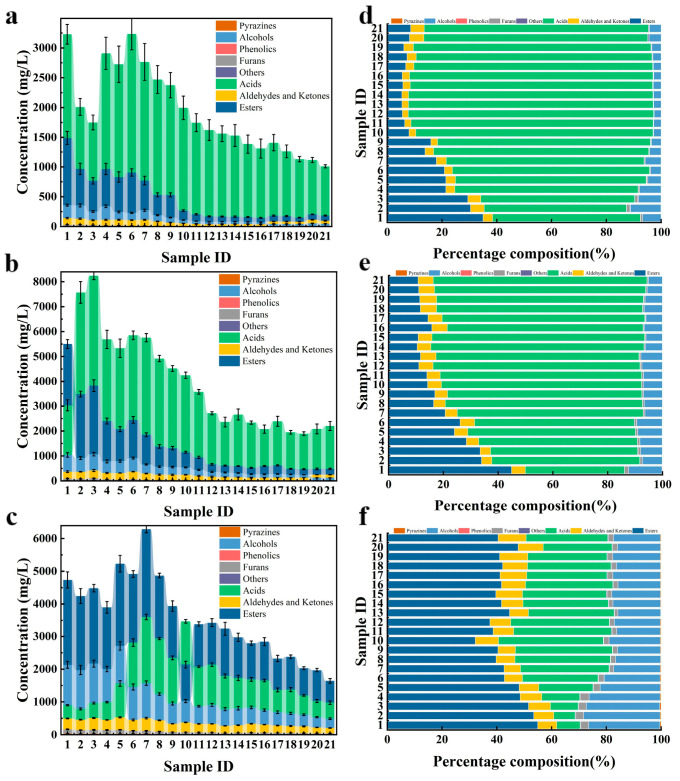
Dynamics of volatile flavor compound content in low-alcohol base baijiu: (**a**) Third round; (**b**) Fourth round; (**c**) Fifth round. Dynamics of percentage of volatile flavor compound content in low-alcohol base baijiu: (**d**) Third round; (**e**) Fourth round; (**f**) Fifth round.

Volatile compound analysis identified eight chemical classes, esters, acids, alcohols, aldehydes and ketones, furans, pyrazines, phenols, and others in low-alcohol base baijiu, revealing clear category-level dynamics during distillation (Figure 3). Across all three rounds, decreasing alcohol strength coincided with a marked rise in the relative proportion of acids and concomitant declines in esters and alcohols, reflecting a shift toward acid enrichment in later stages. Specifically, acids increased from 53.00% to 84.80% (third round), 45.00% to 80.60% (fourth), and 17.29% to 41.70% (fifth) as alcohol levels dropped from >20% (*v*/*v*) to <10% (*v*/*v*). Conversely, esters decreased from 32.50% to 8.46% (third), 39.50% to 12.86% (fourth), and 49.00% to 42.30% (fifth); alcohols declined from 9.00% to 3.40% (third), 9.50% to 6.57% (fourth), and 23.71% to 16.30% (fifth). Unlike typical Jiang-flavor base baijiu where esters dominate followed by alcohols [44], low-alcohol base baijiu was characterized by acid predominance.

Despite the consistent trend, there are significant differences in ester and alcohol retention capacity across rounds, constituting a key dimension of round evolution. The third round showed low initial ester and alcohol proportions that declined sharply with decreasing alcohol strength. The fourth round maintained a relatively stable ester proportion amid higher overall abundance. In contrast, the fifth round retained consistently high ester and alcohol proportions throughout distillation, preserving a combined 58.60% even in the <10% (*v*/*v*) phase, markedly above earlier rounds. Against the background of the common changes driven by the alcohol content, the different rounds showed an enhancement process from acid dominance to esters and alcohols, reflecting the overall evolutionary characteristics of the metabolic system gradually maturing and the accumulation of flavor compounds tending to be stabilized during the brewing process.

Consequently, the changes in the compound composition of low-alcohol base baijiu are essentially the result of the microbial metabolic basis determining compound production, while the distillation process influences the distillation order and enrichment degree of each type of compound [45]. The evolution from third round to fifth round marks the development of the distillation system from the primary metabolic stage, which is dominated by acid production, to the mature and stable stage, which is characterized by the synthesis of complex flavor esters and alcohols. From the harmonious aroma at a high alcohol level to the prominence of off-flavors (sour, salt, etc.) at a low alcohol level, the aromatic composition in the distillation process gradually transforms from a harmonious of enrichment of high saturation to the low saturation of a single compound. This provides a key scientific basis for understanding the formation mechanism of the flavor differences in Jiang-flavor baijiu with low-alcohol base baijiu.

#### 3.3.2. Characteristic Compound Analysis for Flavor Structure Classification of Low-Alcohol Base Baijiu

**Figure 4:**
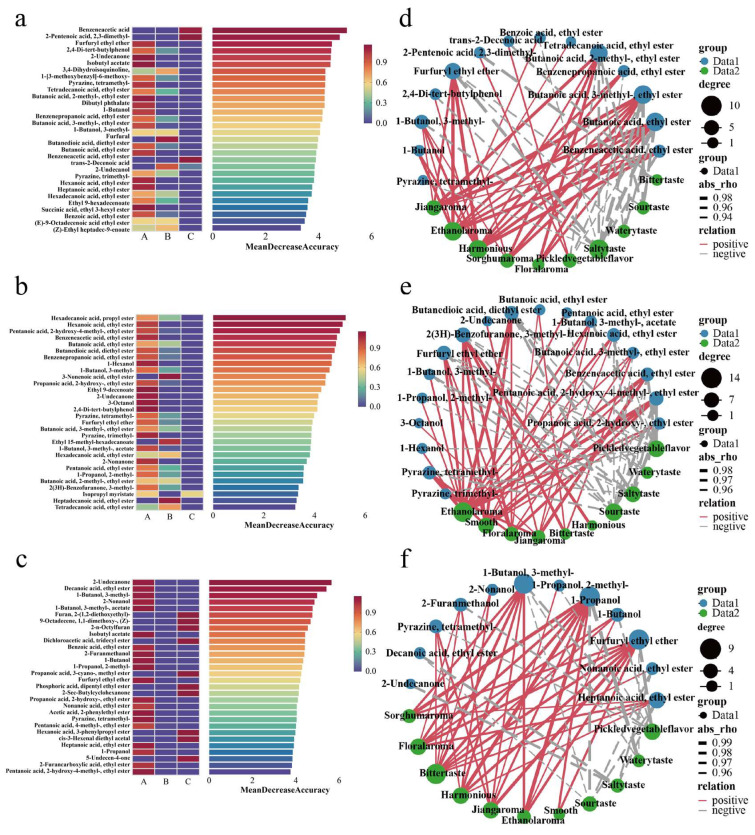
Screening of characteristic flavor compounds for the flavor structure of low-alcohol base baijiu in Random Forest (A: first level; B: second level; C: third level): (**a**) Third round; (**b**) Fourth round; (**c**) Fifth round. Characteristic difference compounds of flavor structure delineation and sensory correlation analysis for low-alcohol base baijiu: (**d**) Third round; (**e**) Fourth round; (**f**) Fifth round.

First, we constructed a Random Forest model to discriminate the hierarchical flavor structure of low-alcohol base baijiu and, within each round, selected stage-difference compounds using variable-importance metrics (Mean Decrease Accuracy and Mean Decrease Gini) [46]. Building on the chemical fingerprints of flavor hierarchies, we correlated screened compounds with sensory attributes to analyze the sensory expression mechanisms and key driving pathways behind the hierarchical differences among rounds of low-alcohol base baijiu. Random Forest results (Figure 4a–c) revealed both cross-round commonalities and round-dependent variations in characteristic compounds. 1-Butanol, 3-methyl-, 2-Undecanone, Furfuryl ethyl ether, and Pyrazine, tetramethyl- can be regarded as stable characteristic compounds for hierarchical classification. However, the discriminatory contributions of these substances were not constant across rounds, but showed significant round-dependent migration. 2-Undecanone jumped from the fifth most important feature in the third round to the first most important feature in the fifth round, a migration that implies a systematic amplification of the role of ketones in flavor stratification as rounds progressed; 1-Butanol, 3-methyl- moved from the fifteenth position in the third round to the third position in the fifth round, suggesting that higher alcohols became more critical flavor differentiators in the maturation round; Furfuryl ethyl ether had a high weight in the third rotation (3rd position), but shifted back in the order in the fourth and fifth rotations (17th and 16th positions), suggesting its role as an early rotation characterization marker; Pyrazine, tetramethyl-distinction tended to weaken with advancing rounds (8th in the third round → 22nd in the fifth round), possibly related to its stabilization in concentration in later rounds, when it ceased to be a major source of variation. This phenomenon of the round-dependent migration of marker importance is another important finding of this study. It reveals that the center of gravity of the flavor differentiation system is not static during the brewing process of white wine but undergoes a systematic feature shift with microbial community succession and metabolite accumulation. This provides a new chemical indicator for the dynamic monitoring of the brewing process.

Inter-round differential features further reflected evolving flavor-classification pathways. The third round was marked by higher rankings of more acidic and unsaturated acid-related products (e.g., Benzeneacetic acid; 2-Pentenoic acid, 2,3-dimethyl-; trans-2-Decenoic acid; Furfural), indicating greater sensitivity to acid production. The fourth round focused on esters (e.g., Hexanoic acid, ethyl ester; Butanedioic acid, diethyl ester; Benzeneacetic acid, ethyl ester), suggesting that the flavor structure classification was mainly dominated by changes in the ester skeleton composition and proportion. The fifth round exhibited a more integrated pattern, featuring additional alcohols and furan derivatives (e.g., 2-Nonanol; 2-Furanmethanol; 2-n-Octylfuran) alongside esters, together with the heightened importance of 2-Undecanone. This suggests that hierarchical differences in higher rounds arise from the broader structural reconfiguration of the flavor matrix rather than a single dominant compound.

After obtaining the characteristic differential compounds from the Random Forest screen described above, they were further correlated with sensory attributes to elucidate how the chemical fingerprints were reflective of the sensory expression of positive aroma versus negative off-flavors (Figure 4d–f). The results showed that 1-Butanol, 3-methyl-, Furfuryl ethyl ether, and Pyrazine, tetramethyl- exhibited stable positive correlations (r > 0.90) with positive sensory attributes (harmonious, smooth, jiang-aroma) and stable negative correlations with negative attributes (salty, sour, watery) in all rounds (r < −0.90). This pattern of stable correlations across rounds suggests that these three classes of compounds are not only statistical discriminatory markers, but also functional flavor modifiers as their presence is beneficial in maintaining positive flavor expression and suppressing off-flavor perception. Of particular interest is Furfuryl ethyl ether; its correlation coefficients were as high as 0.984 with jiang-aroma and −0.984 with pickled vegetable flavor in five rounds, showing strong effect specificity. This near-perfect positive and negative correlation implies that it may directly inhibit the expression of pickled vegetable flavor through some antagonistic mechanism at the perceptual level. This hypothesis deserves subsequent validation by means of molecular sensory science. Among the round-specific compounds, trans-2-Decenoic acid and 2-Pentenoic acid, 2,3-dimethyl- showed a significant positive correlation with salt–cabbage flavor in three rounds, suggesting that these unsaturated acids might be the chemical source of the pickled vegetable flavor burst in the later part of three rounds. In contrast, similar associations disappeared in the fourth and fifth rounds, which may be related to the flavor masking of esters in these rounds.

#### 3.3.3. OAV Analysis of Characteristic Flavor Compounds for Flavor Structure Classification of Low-Alcohol Base Baijiu

**Figure 5:**
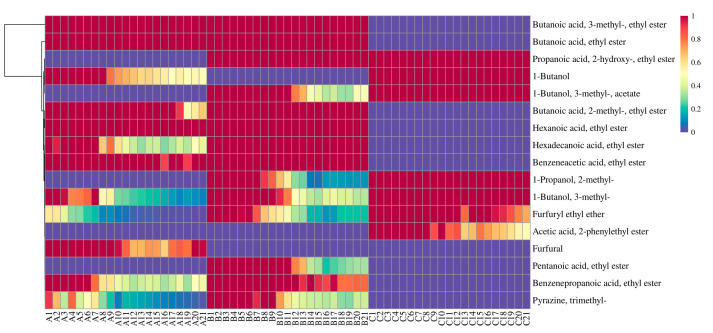
Heat map of OAVs for compounds with OAV > 1 in three rounds (A: Third round; B: Fourth round; C: Fifth round).

To further validate the contribution of the characteristic difference compounds to flavor structure classification in low-alcohol base baijiu, we calculated odor activity values (OAVs) for the selected compounds (Tables S2–S4). Seventeen compounds with OAV > 1 were identified, and an OAV-based clustered heatmap was generated to visualize their variation across samples (Figure 5).

Across all rounds, the OAVs of key aroma-active compounds exhibited strong dependence on alcohol strength, showing a stepwise decline in sensory contribution as alcohol content decreased from >20% (*v*/*v*) to <10% (*v*/*v*) across stages. At high alcohol strength, each round was dominated by signature esters or ester–alcohol combinations that formed the core flavor skeleton and imparted pronounced fruity and sweet notes [25]. When alcohol content dropped to 10–20% (*v*/*v*), the OAVs of the main compounds usually went down, but they often stayed above the sensory thresholds. At the same time, some thermal or Maillard products, such as furfural and furfuryl ethyl ether, appeared or became stronger, and they added toasted and caramel-like notes [47]. Moving into the lower alcohol levels (<10% *v*/*v*), the overall aromatic intensity of all rounds was significantly weakened by a significant reduction in the OAV of the supporting flavor compounds, resulting in a relative accentuation of acidity, a general decline in harmonious characteristics, and a tendency towards a weakening of the structure of the baijiu.

Differences between rounds reveal profound shifts in flavor composition and intensity of the brewing process. The third round represents a relatively early stage, where the flavor skeleton is dominated by simple butanoic acid esters (e.g., butanoic acid, 3-methyl-, ethyl ester) with relatively low and rapidly decaying OAVs, and the caramel signal (furfural) appears only at a later stage, resulting in a monolithic and unstable flavor structure. The fourth round exhibits peak flavor intensity and structural optimization, with significantly higher OAVs for signature butyrate esters (especially Butanoic acid, 3-methyl-, ethyl ester) than the third round, imparting a fuller, richer, sweeter fruity aroma [24], while the inclusion of Propanoic acid, 2-hydroxy-, ethyl ester enriches aroma layers. The fifth round undergoes a fundamental flavor transition: Dominant compounds shift from butyric acid esters to 1-Butanol, 3-methyl-, acetate, Acetic acid, 2-phenylethyl ester, and 1-Butanol, and the aroma profile shifts from the rich fruity-sweetness of earlier rounds toward more elegant banana, rose, and grassy notes [25]. Although some compounds may have lower OAVs than in the fourth round, the complex maturing aroma represented by Furfuryl ethyl ether persists throughout distillation, reflecting a more complex and harmonized flavor composition.

Chemical concentration gradients and compositional variation provide a material basis for sample classifiability; however, their actual contributions to discrimination performance and predictive accuracy require quantitative evaluation. Therefore, we developed and compared multi-class machine learning models to quantify the discriminative utility of individual compounds to classify low-alcohol base baijiu and to assess model robustness across datasets from multiple rounds [48].

### 3.4. Machine Learning-Based Flavor Structure Prediction for Low-Alcohol Base Baijiu

#### 3.4.1. Machine Learning-Based Model Screening for Flavor Structure Prediction of Low-Alcohol Base Baijiu

**Figure 6:**
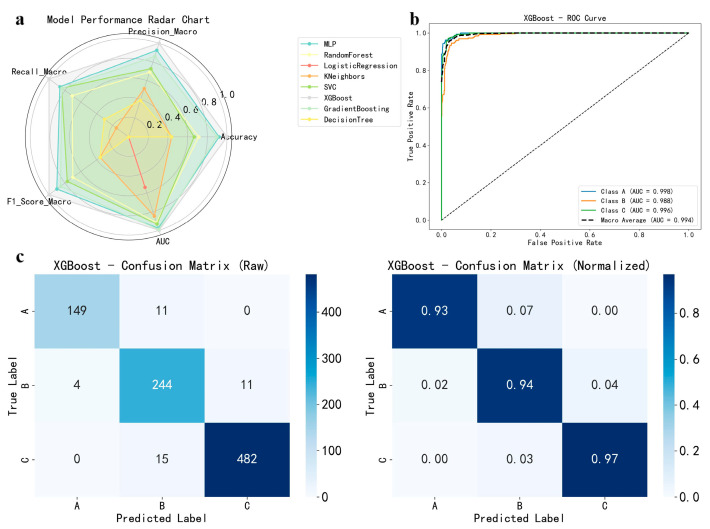
(**a**) Best machine learning model screening for flavor structure of low-alcohol base baijiu. (**b**) ROC curve of best machine learning model for flavor structure segmentation of low-alcohol base baijiu. (**c**) Confusion matrix of best machine learning model for low-alcohol base baijiu flavor structure segmentation (A: stage I; B: stage II; C: stage III).

Using concentrations of volatile flavor compounds as input features, we developed multi-class classifiers to predict the flavor structure stages of low-alcohol base baijiu across three rounds, and compared the performance of eight models including the MLPClassifier, DecisionTreeClassifier, LogisticRegression, RandomForestClassifier, XGBClassifier, and SVC (Figure 6). Model performance differed substantially. Overall, XGBoost achieved the best results, ranking first for Accuracy (0.95), Precision (0.95), Recall (0.94), F1 (0.95), and AUC (0.99), indicating excellent overall classification capability. The AUC approaching 0.994 further suggests strong discriminative power across classes, supporting the use of XGBoost for subsequent flavor quality analysis and prediction (Figure 6a). Consistently, ROC curves for all classes showed areas under the curve > 0.95 (Figure 6b), and the confusion matrix indicated that classification accuracy exceeded 90% for all predicted classes in the test set (Figure 6c).

First, the XGBoost AUC value of up to 0.9939 proves that the dataset consisting of flavor compounds indeed contains nonlinear boundaries corresponding to the sensory three-stage model that can be accurately captured by the algorithm. This validates the objectivity of our proposed three-stage flavor structure model at the chemical level; if the three-stage division of sensory qualities was artificial and arbitrary, it is unlikely that the machine learning model could achieve such a high prediction accuracy. Secondly, XGBoost, as a tree integration model based on gradient boosting, can capture the subtle and nonlinear patterns in the data layer by layer, and thus more finely delineate the complex differences in the composition of flavor compounds among different rounds of low-alcohol base baijiu [49]. Third, the confusion matrix shows (Figure 6c) that the model’s prediction errors were mainly concentrated between adjacent stages (e.g., misclassifying stage II as stage I or stage III), while there were almost no cross-stage misclassifications (e.g., misclassifying stage I as stage III). This suggests that the boundary between the three stages is a fuzzy continuous transition rather than a truncated separation, which is consistent with the physical nature of the flavor gradient in the actual brewing process.

#### 3.4.2. Shap Characterization

**Figure 7:**
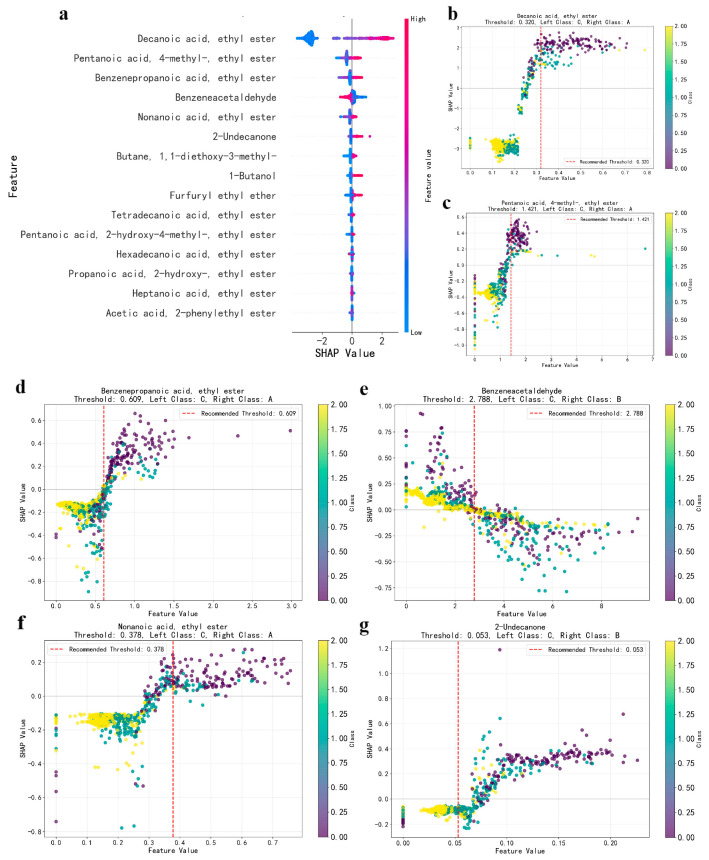
(**a**) SHAP bar chart showing the order of characteristic importance of the compounds in the flavor structure prediction model for low-alcohol base baijiu. Characteristic compounds dependency analysis based on SHAP analysis: (**b**) Decanoic acid, ethyl ester; (**c**) Pentanoic acid, 4-methyl, ethyl ester; (**d**) Benzenepropanoic acid, ethyl ester; (**e**) Benzeneacetaldehyde; (**f**) Nonanoic acid, ethyl ester; (**g**) 2-Undecanone.

To improve model credibility and transparency [50], we applied SHAP to the best-performing model to rank and interpret the top 15 flavor-related features (Figure 7). Across the three rounds of XGBoost-based flavor structure classification, SHAP results highlighted Decanoic acid, ethyl ester (Figure 7b) as a highly discriminative marker, with a differentiation score of 4.26, high SHAP importance, and a low recommended threshold (0.32 mg/L). This indicates that even trace-level variation in this compound can strongly and directionally influence model predictions, making it among the most sensitive markers for discriminating low-alcohol base baijiu quality. Similarly, Heptanoic acid, ethyl ester (Figure S1h; 0.003 mg/L) and Nonanoic acid, ethyl ester (Figure 7f; 0.38 mg/L) exhibited low thresholds and high sensitivity, together constituting a set of rapidly responsive indicators within the low-alcohol base baijiu flavor matrix.

Beyond feature ranking, SHAP also revealed distinct contribution patterns across compounds. For example, Benzeneacetaldehyde (Figure 7e) and 2-Undecanone (Figure 7g) have different dominant categories on either side of the threshold and opposite signs of the SHAP means, suggesting that when their concentrations exceed the thresholds (2.79 mg/L and 0.05 mg/L, respectively), the contribution to the model shifts from positive to negative. This reversal of action, which in moderation contributes pleasant floral notes and in excess may lead to undesirable flavors, clearly points to its two-fold nature in flavor composition. In contrast, Propanoic acid, 2-hydroxy-, ethyl ester (Figure S1g) had an unusually high recommended threshold (3896.44 mg/L), and the magnitude of variation in both its differentiation score and SHAP mean was small. This confirms that it is a foundational flavor component in low-alcohol base baijiu, present in high concentrations, but its concentration fluctuations have limited contribution to model differentiation of different samples. Pentanoic acid, 2-hydroxy-4-methyl-, ethyl ester (Figure S1e) (15.59 mg/L) and Hexadecanoic acid, ethyl ester (Figure S1f) (28.42 mg/L) showed similar high threshold, low discrimination characteristics.

Notably, OAV analysis and machine learning models reveal two different dimensions of flavor compounds; OAV reflects a compound’s absolute sensory contribution potential—i.e., how strong a sensory effect it can have if left alone. Machine learning (specifically SHAP) reflects the relative discriminatory contribution of a compound—i.e., how much its concentration variation contributes to differentiating between samples in complex matrices. These two dimensions do not always agree. For example, Decanoic acid, ethyl ester did not have the highest OAV (28.5 in the three rounds of stage I), yet it was the most important discriminant feature in the SHAP analysis (Figure 7). This suggests that the flavor importance of a compound depends not only on its own aroma intensity, but also on its degree of inter-sample variability and its interactions with other substances. Traditional OAV analysis is unable to capture this information weighting, which is the unique advantage of machine learning methods.

The above SHAP analyses not only screened out the key compounds affecting the flavor quality of low-alcohol base baijiu, but also quantitatively depicted the inflection points and patterns of their influences through the recommended thresholds and differentiation levels. This indicates that in production practice, the focus of monitoring and control should be on low-threshold and highly sensitive esters such as Decanoic acid, ethyl ester, etc., and their concentrations should be strictly controlled to prevent them from triggering negative flavors. For high threshold compounds such as Propanoic acid, 2-hydroxy-, ethyl ester, their concentration range is wider and the urgency of process control is relatively low.

## 4. Concludes

Based on sensory and flavor analysis, a three-stage flavor structure model was developed for low-alcohol base baijiu of the Jiang-flavor baijiu (>20% vol, 10–20% vol, <10% vol). Accurate prediction of flavor stages was achieved by the XGBoost model (F1 0.9494, AUC 0.9939), and flavor regulation nodes were proposed based on the SHAP analysis, which classified the key compounds into high-sensitivity nodes (e.g., Decanoic acid, ethyl ester, with a threshold value of 0.8 mg/L), bidirectional-action nodes (e.g., Benzeneacetaldehyde, which exhibits a two-sided nature of beneficial at low concentration and harmful at high concentration) and base background nodes (e.g., Propanoic acid, 2-hydroxy-, ethyl ester). This study provides a systematic scientific basis and quantifiable regulatory targets for the quality grading, precise blending and high value utilization of low-alcohol base baijiu.

## Data Availability

The data presented in this study are available from the corresponding author upon reasonable request. Due to confidentiality agreements and commercial restrictions associated with an industry collaborative project, the raw data are not publicly available. Relevant data supporting the findings of this study can be provided by the corresponding author after permission from the cooperating company.

## Supplementary Materials

The following supporting information can be downloaded at: https://www.mdpi.com/article/10.3390/foods15111891/s1, Table S1. Standard curve for flavor compounds. Table S2. OAV table for third rounds of characteristic compounds (“-” indicates that this compound was not detected). Table S3. OAV table for fourth rounds of characteristic compounds (“-” indicates that this compound was not detected). Table S4. OAV table for fifth rounds of characteristic compounds (“-” indicates that this compound was not detected). Figure S1. Characteristic compounds dependency analysis based on SHAP analysis.

## Abbreviations

The following abbreviations are used in this manuscript:

SHAP	Shapley Additive Explanations
OAV	Odor Activity Value
