# Supplementary material for: Decoding the Flavor Structure of Jiang-Flavor Low-Alcohol Base Baijiu: A Machine Learning-Driven Approach to Reveal the Flavor Evolution Patterns and Key Quality Control Nodes
Source: Foods. 2026 May 27;15(11):1891. doi: 10.3390/foods15111891 (PMC13257113; doi:10.3390/foods15111891)
Supplement: Supplementary file 1 [file foods-15-01891-s001.zip › foods-4239241-supplementary.pdf]

Supplementary Materials

Table S1. Standard curve for flavor compounds

No.	CAS	Flavor components	Standard curve	No.	CAS	Flavor components	Standard curve
1	14667-55-1	Pyrazine, trimethyl-	y = 18.747x - 0.0073	28	538-68-1	Benzene, pentyl-	y = 0.0365x + 0.0045
2	96-76-4	2,4-Di-tert-butylphenol	y = 0.0793x + 0.0031	29	539-82-2	Pentanoic acid, ethyl ester	y = 1.2981x - 0.0005
3	13019-16-4	2-Octenal, 2-butyl-	y = 0.0388x + 0.0119	30	106-32-1	Octanoic acid, ethyl ester	y = 0.0504x + 0.0154
4	821-55-6	2-Nonanone	y = 0.1869x - 0.0006	31	544-35-4	Linoleic acid ethyl ester	y = 5.1229x - 0.0441
5	2345-28-0	2-Pentadecanone	y = 0.0198x - 0.0016	32	103-45-7	Acetic acid, 2-phenylethyl ester	y = 0.7552x - 0.0015
6	112-12-9	2-Undecanone	y = 0.0411x + 0.0036	33	110-19-0	Isobutyl acetate	y = 3.2477x - 0.003
7	3777-69-3	Furan, 2-pentyl-	y = 0.0915x + 0.0278	34	123-92-2	1-Butanol, 3-methyl-, acetate	y = 1.7321x - 0.0007
8	1193-79-9	2-Acetyl-5-methylfuran	y = 12.242x - 0.0056	35	78-83-1	1-Propanol, 2-methyl-	y = 138.65x + 0.1093
9	2021-28-5	Benzenepropanoic acid, ethyl ester	y = 0.3188x - 0.0006	36	25415-67-2	Pentanoic acid, 4-methyl-, ethyl ester	y = 0.4729x + 0.0509
10	108-64-5	Butanoic acid, 3-methyl-, ethyl ester	y = 1.4301x + 0.0173	37	123-51-3	1-Butanol, 3-methyl-	y = 26.453x + 0.0512
11	10348-47-7	Pentanoic acid, 2-hydroxy-4-methyl-, ethyl ester	y = 13.929x - 0.0005	38	503-74-2	Butanoic acid, 3-methyl-	y = 59.139x + 0.283
12	100-52-7	Benzaldehyde	y = 1.9457x - 0.0036	39	106-33-2	Dodecanoic acid, ethyl ester	y = 0.0277x - 0.0007
13	60-12-8	Phenylethyl Alcohol	y = 18.728x - 0.0045	40	71-36-3	1-Butanol	y = 74.516x + 0.1959
14	64-19-7	Acetic acid	y = 518.7x + 0.458	41	502-69-2	2-Pentadecanone, 6,10,14-trimethyl-	y = 0.039x - 0.0002
15	71-23-8	1-Propanol	y = 746.63x + 0.2438	42	3658-80-8	Dimethyl trisulfide	y = 0.6699x + 0.1347
16	2305-05-7	γ-Dodecalactone	y = 0.3106x + 0.0338	43	620-02-0	2-Furancarboxaldehyde, 5-methyl-	y = 10.286x - 0.016
17	1124-11-4	Pyrazine, tetramethyl-	y = 11.01x - 0.0081	44	122-78-1	Benzeneacetaldehyde	y = 2.9949x - 0.0071
18	105-54-4	Butanoic acid, ethyl ester	y = 3.4332x + 0.0268	45	6314-97-2	Benzene, (2,2-diethoxyethyl)-	y = 0.474x + 0.0134
19	106-30-9	Heptanoic acid, ethyl ester	y = 0.1791x + 0.0088	46	10031-93-3	Benzenebutanoic acid, ethyl ester	y = 0.1729x - 0.002
20	110-38-3	Decanoic acid, ethyl ester	y = 0.0262x + 0.0133	47	14010-23-2	Heptadecanoic acid, ethyl ester	y = 1.8935x - 0.0564
21	123-66-0	Hexanoic acid, ethyl ester	y = 0.4547x + 0.0073	48	6270-56-0	Furfuryl ethyl ether	y = 19.882x - 0.0019
22	98-01-1	Furfural	y = 14.616x - 0.0046	49	91-20-3	Naphthalene	y = 0.3355x - 0.0021
23	624-17-9	Diethyl azelate	y = 0.6688x + 0.0206	50	21834-92-4	5-Methyl-2-phenyl-2-hexenal	y = 1.1094x + 0.0009
24	123-29-5	Nonanoic acid, ethyl ester	y = 0.044x + 0.0149	51	3433-16-7	Nonanoic acid, 9-oxo-, ethyl ester	y = 16.769x + 0.0047
25	97-64-3	Propanoic acid, 2-hydroxy-, ethyl ester	y = 152.51x + 0.2047	52	123-07-9	Phenol, 4-ethyl-	y = 17.525x - 0.0005
26	28267-29-0	Ethyl tridecanoate	y = 0.0173x - 0.0002	53	628-97-7	Hexadecanoic acid, ethyl ester	y = 0.5387x - 0.0028
27	124-06-1	Tetradecanoic acid, ethyl ester	y = 0.0226x + 0.0089	54	84-74-2	Dibutyl phthalate	y = 4.3512x + 0.0105

Table S2. OAV table for third rounds of characteristic compounds ("-" indicates that this compound was not detected)

Flavor components	CAS	Threshold(mg/L)	Aroma Description	25.23%vol	22.47%vol	18.33%vol	15.93%vol	13.3%vol	11.37%vol	9.67%vol	8.13%vol	6.67%vol	5.60%vol	4.63%vol
1-Butanol, 3-methyl-	123-51-3	70.00	fruit, flower, foul odor	1.49	1.79	<1	<1	<1	<1	1.07	<1	<1	<1	<1
Propanoic acid, 2-hydroxy-, ethyl ester	97-64-3	128.08	sweet, fruit, grass	-	-	-	-	-	-	-	-	-	-	-
Furfuryl ethyl ether	6270-56-0	40.87	caramel, toasted bread	<1	<1	<1	<1	<1	<1	<1	<1	<1	<1	<1
1-Propanol, 2-methyl-	78-83-1	28.26	fusel oil, malt, wine	-	-	-	-	-	-	-	-	-	-	-
Hexadecanoic acid, ethyl ester	628-97-7	8.54	waxy, butter	3.18	<1	1.62	1.71	1.97	1.69	1.40	<1	<1	<1	<1
1-Butanol	71-36-3	2.73	fruit	6.66	3.59	2.94	2.79	2.40	2.04	1.81	1.26	<1	<1	<1
Benzeneacetic acid, ethyl ester	101-97-3	0.41	flower, fruit, banana	8.10	6.74	4.92	4.47	3.77	3.17	3.05	2.43	1.39	1.44	1.33
Benzenepropanoic acid, ethyl ester	2021-28-5	0.13	sweet, flower	2.51	1.79	1.20	1.31	1.25	1.06	<1	<1	<1	<1	<1
1-Butanol, 3-methyl-, acetate	123-92-2	0.09	banana, sweet, fruit candy	-	-	-	-	-	-	-	-	-	-	-
Butanoic acid, ethyl ester	105-54-4	0.08	apple, pineapple, fruit, floral	45.42	44.68	35.95	31.30	30.11	25.63	25.01	18.34	10.66	11.53	10.32
Butanoic acid, 2-methyl-, ethyl ester	7452-79-1	0.06	pineapple, apple, sweet	20.96	20.59	21.62	15.98	14.31	15.07	16.76	10.70	5.13	5.94	4.50
Hexanoic acid, ethyl ester	123-66-0	0.06	sweet, fruit, cellar, cucumber	15.57	13.77	10.47	10.06	9.43	6.78	5.45	4.26	2.08	5.61	9.44
Butanoic acid, 3-methyl-, ethyl ester	108-64-5	0.01	apple, pineapple, banana, fruit	762.94	824.01	675.79	619.24	573.64	499.36	459.84	324.18	217.46	216.49	182.44
Furfural	98-01-1	44.03	burnt, nutty, rancid	2.32	2.18	1.79	2.01	2.00	2.01	2.15	1.58	1.23	<1	<1
Acetic acid, 2-phenylethyl ester	103-45-7	0.91	flower, rose	-	-	-	-	-	-	-	-	-	-	-
Pyrazine, trimethyl-	14667-55-1	0.73	green pepper, coffee, toasted bread	<1	<1	<1	<1	<1	<1	<1	<1	<1	<1	<1
Pentanoic acid, ethyl ester	539-82-2	0.03	fruit, cheese	-	-	-	-	-	-	-	-	-	-	-
Butanedioic acid, diethyl ester	123-25-1	353.19	fruit, flower, pollen	<1	<1	<1	<1	<1	<1	<1	<1	<1	<1	<1
1-Propanol	71-23-8	314.00	fruit, flower, grass	-	-	-	-	-	-	-	-	-	-	-
Pyrazine, tetramethyl-	1124-11-4	80.07	sweet, fruit	<1	<1	<1	<1	<1	<1	<1	<1	<1	<1	<1
Heptanoic acid, ethyl ester	106-30-9	13.15	flower, fruit, honey, sweet	<1	<1	<1	<1	<1	<1	<1	<1	<1	<1	<1
1-Hexanol	111-27-3	5.30	grass, resin, cream	-	-	-	-	-	-	-	-	-	-	-
Nonanoic acid, ethyl ester	123-29-5	3.15	ester, honey, fruit	-	-	-	-	-	-	-	-	-	-	-
2-Furanmethanol	98-00-0	2.00	caramel, toast, burnt rubber	-	-	-	-	-	-	-	-	-	-	-
Benzoic acid, ethyl ester	93-89-0	1.43	rose	<1	<1	<1	<1	<1	<1	<1	<1	<1	<1	<1
Decanoic acid, ethyl ester	110-38-3	1.12	pineapple, fruit, flower	-	-	-	-	-	-	-	-	-	-	-
Isobutyl acetate	110-19-0	0.92	apple, banana	<1	<1	<1	<1	<1	<1	<1	<1	<1	<1	<1
Tetradecanoic acid, ethyl ester	124-06-1	0.50	sweet, waxy	<1	<1	<1	<1	<1	<1	<1	<1	<1	<1	<1

Table S2. Continued. OAV table for third rounds of characteristic compounds (“-“ indicates that this compound was not detected)

Flavor components	CAS	Threshold(mg/L)	Aroma Description	3.87%vol	2.93%vol	2.43%vol	1.93%vol	1.57%vol	1.23%vol	1.07%vol	0.80%vol	0.67%vol	0.50%vol
1-Butanol, 3-methyl-	123-51-3	70.00	fruit, flower, foul odor	<1	<1	<1	<1	<1	<1	<1	<1	<1	<1
Propanoic acid, 2-hydroxy-, ethyl ester	97-64-3	128.08	sweet, fruit, grass	-	-	-	-	-	-	-	-	-	-
Furfuryl ethyl ether	6270-56-0	40.87	caramel, toasted bread	<1	<1	<1	<1	<1	<1	<1	<1	<1	<1
1-Propanol, 2-methyl-	78-83-1	28.26	fusel oil, malt, wine	-	-	-	-	-	-	-	-	-	-
Hexadecanoic acid, ethyl ester	628-97-7	8.54	waxy, butter	<1	<1	<1	<1	<1	<1	<1	<1	<1	<1
1-Butanol	71-36-3	2.73	fruit	<1	<1	<1	<1	<1	<1	<1	<1	<1	<1
Benzeneacetic acid, ethyl ester	101-97-3	0.41	flower, fruit, banana	1.06	1.13	1.07	1.01	<1	1.02	1.00	<1	1.24	1.11
Benzenepropanoic acid, ethyl ester	2021-28-5	0.13	sweet, flower	<1	<1	<1	<1	<1	<1	<1	<1	<1	<1
1-Butanol, 3-methyl-, acetate	123-92-2	0.09	banana, sweet, fruit candy	-	-	-	-	-	-	-	-	-	-
Butanoic acid, ethyl ester	105-54-4	0.08	apple, pineapple, fruit, floral	7.66	6.83	6.26	5.73	5.09	5.02	4.65	4.08	4.19	4.37
Butanoic acid, 2-methyl-, ethyl ester	7452-79-1	0.06	pineapple, apple, sweet	2.84	2.40	1.82	1.55	1.17	1.12	<1	<1	<1	<1
Hexanoic acid, ethyl ester	123-66-0	0.06	sweet, fruit, cellar, cucumber	7.17	6.65	6.29	5.77	5.36	4.96	2.90	2.59	2.39	1.83
Butanoic acid, 3-methyl-, ethyl ester	108-64-5	0.01	apple, pineapple, banana, fruit	125.88	110.40	90.05	80.05	66.96	65.73	60.28	43.95	53.32	51.93
Furfural	98-01-1	44.03	burnt, nutty, rancid	<1	<1	<1	<1	<1	<1	<1	<1	1.17	<1
Acetic acid, 2-phenylethyl ester	103-45-7	0.91	flower, rose	-	-	-	-	-	-	-	-	-	-
Pyrazine, trimethyl-	14667-55-1	0.73	green pepper, coffee, toasted bread	<1	<1	<1	<1	<1	<1	<1	<1	<1	<1
Pentanoic acid, ethyl ester	539-82-2	0.03	fruit, cheese	-	-	-	-	-	-	-	-	-	-
Butanedioic acid, diethyl ester	123-25-1	353.19	fruit, flower, pollen	<1	<1	<1	<1	<1	<1	<1	<1	<1	<1
1-Propanol	71-23-8	314.00	fruit, flower, grass	-	-	-	-	-	-	-	-	-	-
Pyrazine, tetramethyl-	1124-11-4	80.07	sweet, fruit	<1	<1	<1	<1	<1	<1	<1	<1	<1	<1
Heptanoic acid, ethyl ester	106-30-9	13.15	flower, fruit, honey, sweet	<1	<1	<1	<1	<1	<1	<1	<1	<1	<1
1-Hexanol	111-27-3	5.30	grass, resin, cream	-	-	-	-	-	-	-	-	-	-
Nonanoic acid, ethyl ester	123-29-5	3.15	ester, honey, fruit	-	-	-	-	-	-	-	-	-	-
2-Furanmethanol	98-00-0	2.00	caramel, toast, burnt rubber	-	-	-	-	-	-	-	-	-	-
Benzoic acid, ethyl ester	93-89-0	1.43	rose	<1	<1	<1	<1	<1	<1	<1	<1	<1	<1
Decanoic acid, ethyl ester	110-38-3	1.12	pineapple, fruit, flower	-	-	-	-	-	-	-	-	-	-
Isobutyl acetate	110-19-0	0.92	apple, banana	<1	<1	<1	<1	<1	<1	<1	<1	<1	<1
Tetradecanoic acid, ethyl ester	124-06-1	0.50	sweet, waxy	<1	<1	<1	<1	<1	<1	<1	<1	<1	<1

Table S3. OAV table for fourth rounds of characteristic compounds ("-" indicates that this compound was not detected)

Flavor components	CAS	Threshold(mg/L)	Aroma Description	28.87%vol	22.27%vol	17.83%vol	15.23%vol	13.83%vol	12.47%vol	11.10%vol	9.77%vol	8.93%vol	8.17%vol	6.87%vol
1-Butanol, 3-methyl-	123-51-3	70.00	fruit, flower, foul odor	3.03	2.29	2.86	2.30	1.65	1.85	1.39	1.09	<1	<1	<1
Propanoic acid, 2-hydroxy-, ethyl ester	97-64-3	128.08	sweet, fruit, grass	17.16	18.17	16.44	11.05	8.97	10.20	7.82	5.44	4.98	3.97	3.34
Furfuryl ethyl ether	6270-56-0	40.87	caramel, toasted bread	1.95	1.54	1.71	1.23	<1	1.27	<1	<1	<1	<1	<1
1-Propanol, 2-methyl-	78-83-1	28.26	fusel oil, malt, wine	3.05	2.47	3.23	1.74	1.63	1.87	1.22	<1	<1	<1	<1
Hexadecanoic acid, ethyl ester	628-97-7	8.54	waxy, butter	5.76	13.92	22.68	6.89	7.95	7.08	7.31	5.16	4.71	3.18	2.67
1-Butanol	71-36-3	2.73	fruit	-	-	-	-	-	-	-	-	-	-	-
Benzeneacetic acid, ethyl ester	101-97-3	0.41	flower, fruit, banana	16.77	12.18	10.96	7.99	7.41	7.73	6.22	4.89	4.47	4.56	3.84
Benzenepropanoic acid, ethyl ester	2021-28-5	0.13	sweet, flower	4.56	3.05	5.67	2.02	2.13	2.04	1.87	1.55	1.41	1.29	1.08
1-Butanol, 3-methyl-, acetate	123-92-2	0.09	banana, sweet, fruit candy	7.68	3.87	4.40	3.41	3.25	3.15	2.63	1.84	1.63	1.41	1.18
Butanoic acid, ethyl ester	105-54-4	0.08	apple, pineapple, fruit, floral	100.25	90.77	112.73	79.61	72.87	81.87	63.95	45.45	41.55	38.39	32.28
Butanoic acid, 2-methyl-, ethyl ester	7452-79-1	0.06	pineapple, apple, sweet	26.82	27.61	39.96	24.90	22.25	23.81	18.73	13.08	11.95	8.94	7.52
Hexanoic acid, ethyl ester	123-66-0	0.06	sweet, fruit, cellar, cucumber	28.56	26.44	24.81	15.04	12.65	13.13	13.46	7.62	6.96	5.90	5.17
Butanoic acid, 3-methyl-, ethyl ester	108-64-5	0.01	apple, pineapple, banana, fruit	1239.66	1037.2	1295.4	947.90	876.31	936.56	743.28	504.04	460.71	402.77	338.68
Furfural	98-01-1	44.03	burnt, nutty, rancid	-	-	-	-	-	-	-	-	-	-	-
Acetic acid, 2-phenylethyl ester	103-45-7	0.91	flower, rose	-	-	-	-	-	-	-	-	-	-	-
Pyrazine, trimethyl-	14667-55-1	0.73	green pepper, coffee, toasted bread	4.61	3.06	1.71	2.41	1.74	2.26	<1	1.12	1.02	<1	<1
Pentanoic acid, ethyl ester	539-82-2	0.03	fruit, cheese	8.12	4.64	6.78	5.21	4.03	4.35	3.63	2.12	1.94	1.69	1.42
Butanedioic acid, diethyl ester	123-25-1	353.19	fruit, flower, pollen	<1	<1	<1	<1	<1	<1	<1	<1	<1	<1	<1
1-Propanol	71-23-8	314.00	fruit, flower, grass	-	-	-	-	-	-	-	-	-	-	-
Pyrazine, tetramethyl-	1124-11-4	80.07	sweet, fruit	<1	<1	<1	<1	<1	<1	<1	<1	<1	<1	<1
Heptanoic acid, ethyl ester	106-30-9	13.15	flower, fruit, honey, sweet	-	-	-	-	-	-	-	-	-	-	-
1-Hexanol	111-27-3	5.30	grass, resin, cream	<1	<1	<1	<1	<1	<1	<1	<1	<1	<1	<1
Nonanoic acid, ethyl ester	123-29-5	3.15	ester, honey, fruit	-	-	-	-	-	-	-	-	-	-	-
2-Furanmethanol	98-00-0	2.00	caramel, toast, burnt rubber	-	-	-	-	-	-	-	-	-	-	-
Benzoic acid, ethyl ester	93-89-0	1.43	rose	-	-	-	-	-	-	-	-	-	-	-
Decanoic acid, ethyl ester	110-38-3	1.12	pineapple, fruit, flower	-	-	-	-	-	-	-	-	-	-	-
Isobutyl acetate	110-19-0	0.92	apple, banana	-	-	-	-	-	-	-	-	-	-	-
Tetradecanoic acid, ethyl ester	124-06-1	0.50	sweet, waxy	<1	<1	<1	<1	<1	<1	<1	<1	<1	<1	<1

Table S3. Continued. OAV table for fourth rounds of characteristic compounds ("-" indicates that this compound was not detected)

Flavor components	CAS	Threshold(mg/L)	Aroma Description	6.03%vol	5.40%vol	4.83%vol	4.40%vol	4.07%vol	4.47%vol	4.46%vol	4.43%vol	4.39%vol	4.37%vol
1-Butanol, 3-methyl-	123-51-3	70.00	fruit, flower, foul odor	<1	<1	<1	<1	<1	<1	<1	<1	<1	<1
Propanoic acid, 2-hydroxy-, ethyl ester	97-64-3	128.08	sweet, fruit, grass	1.80	1.61	1.42	1.30	1.34	1.47	1.22	1.21	1.43	1.42
Furfuryl ethyl ether	6270-56-0	40.87	caramel, toasted bread	<1	<1	<1	<1	<1	<1	<1	<1	<1	<1
1-Propanol, 2-methyl-	78-83-1	28.26	fusel oil, malt, wine	<1	<1	<1	<1	<1	<1	<1	<1	<1	<1
Hexadecanoic acid, ethyl ester	628-97-7	8.54	waxy, butter	1.64	1.47	1.80	1.64	1.51	1.66	1.52	1.51	1.30	1.29
1-Butanol	71-36-3	2.73	fruit	-	-	-	-	-	-	-	-	-	-
Benzeneacetic acid, ethyl ester	101-97-3	0.41	flower, fruit, banana	3.26	2.92	2.90	2.64	2.47	2.71	2.52	2.50	2.61	2.60
Benzenepropanoic acid, ethyl ester	2021-28-5	0.13	sweet, flower	1.03	<1	1.01	<1	<1	<1	<1	<1	<1	<1
1-Butanol, 3-methyl-, acetate	123-92-2	0.09	banana, sweet, fruit candy	<1	<1	<1	<1	<1	<1	<1	<1	<1	<1
Butanoic acid, ethyl ester	105-54-4	0.08	apple, pineapple, fruit, floral	22.22	19.89	15.88	14.46	13.31	14.62	13.34	13.22	14.71	14.61
Butanoic acid, 2-methyl-, ethyl ester	7452-79-1	0.06	pineapple, apple, sweet	4.63	4.15	3.00	2.74	2.54	2.79	3.17	3.14	2.80	2.78
Hexanoic acid, ethyl ester	123-66-0	0.06	sweet, fruit, cellar, cucumber	4.74	4.27	4.15	3.78	3.31	3.19	2.77	2.45	1.92	1.50
Butanoic acid, 3-methyl-, ethyl ester	108-64-5	0.01	apple, pineapple, banana, fruit	223.90	200.51	147.31	134.19	125.42	137.75	116.66	115.61	141.94	140.97
Furfural	98-01-1	44.03	burnt, nutty, rancid	-	-	-	-	-	-	-	-	-	-
Acetic acid, 2-phenylethyl ester	103-45-7	0.91	flower, rose	-	-	-	-	-	-	-	-	-	-
Pyrazine, trimethyl-	14667-55-1	0.73	green pepper, coffee, toasted bread	<1	<1	<1	<1	<1	<1	<1	<1	<1	<1
Pentanoic acid, ethyl ester	539-82-2	0.03	fruit, cheese	<1	<1	<1	<1	<1	<1	<1	<1	<1	<1
Butanedioic acid, diethyl ester	123-25-1	353.19	fruit, flower, pollen	<1	<1	<1	<1	<1	<1	<1	<1	<1	<1
1-Propanol	71-23-8	314.00	fruit, flower, grass	-	-	-	-	-	-	-	-	-	-
Pyrazine, tetramethyl-	1124-11-4	80.07	sweet, fruit	<1	<1	<1	<1	<1	<1	<1	<1	<1	<1
Heptanoic acid, ethyl ester	106-30-9	13.15	flower, fruit, honey, sweet	-	-	-	-	-	-	-	-	-	-
1-Hexanol	111-27-3	5.30	grass, resin, cream	<1	<1	<1	<1	<1	<1	<1	<1	<1	<1
Nonanoic acid, ethyl ester	123-29-5	3.15	ester, honey, fruit	-	-	-	-	-	-	-	-	-	-
2-Furanmethanol	98-00-0	2.00	caramel, toast, burnt rubber	-	-	-	-	-	-	-	-	-	-
Benzoic acid, ethyl ester	93-89-0	1.43	rose	-	-	-	-	-	-	-	-	-	-
Decanoic acid, ethyl ester	110-38-3	1.12	pineapple, fruit, flower	-	-	-	-	-	-	-	-	-	-
Isobutyl acetate	110-19-0	0.92	apple, banana	-	-	-	-	-	-	-	-	-	-
Tetradecanoic acid, ethyl ester	124-06-1	0.50	sweet, waxy	<1	<1	<1	<1	<1	<1	<1	<1	<1	<1

Table S4. OAV table for fifth rounds of characteristic compounds (“-“ indicates that this compound was not detected)

Flavor components	CAS	Threshold(mg/L)	Aroma Description	38.67%vol	35.27 %vol	32.33 %vol	29.37 %vol	26.23 %vol	23.50 %vol	20.70 %vol	18.2% vol	16.33 %vol	15.07 %vol	13.90 %vol
1-Butanol, 3-methyl-	123-51-3	70.00	fruit, flower, foul odor	6.41	5.84	5.73	4.83	5.31	4.41	4.70	3.50	2.42	2.58	2.07
Propanoic acid, 2-hydroxy-, ethyl ester	97-64-3	128.08	sweet, fruit, grass	19.54	16.12	16.66	14.06	17.61	15.08	18.63	13.91	10.62	7.39	8.03
Furfuryl ethyl ether	6270-56-0	40.87	caramel, toasted bread	3.14	2.92	3.02	2.86	3.04	2.43	2.25	1.97	1.53	1.56	1.32
1-Propanol, 2-methyl-	78-83-1	28.26	fusel oil, malt, wine	11.51	10.55	10.54	8.60	10.40	8.46	9.66	5.40	4.40	4.51	3.21
Hexadecanoic acid, ethyl ester	628-97-7	8.54	waxy, butter	-	-	-	-	-	-	-	-	-	-	-
1-Butanol	71-36-3	2.73	fruit	31.96	26.16	24.72	22.32	26.76	23.53	19.60	12.71	13.06	10.67	11.55
Benzeneacetic acid, ethyl ester	101-97-3	0.41	flower, fruit, banana	-	-	-	-	-	-	-	-	-	-	-
Benzenepropanoic acid, ethyl ester	2021-28-5	0.13	sweet, flower	-	-	-	-	-	-	-	-	-	-	-
1-Butanol, 3-methyl-, acetate	123-92-2	0.09	banana, sweet, fruit candy	50.30	39.07	36.56	29.79	28.92	24.09	20.42	10.25	10.60	8.81	7.55
Butanoic acid, ethyl ester	105-54-4	0.08	apple, pineapple, fruit, floral	-	-	-	-	-	-	-	-	-	-	-
Butanoic acid, 2-methyl-, ethyl ester	7452-79-1	0.06	pineapple, apple, sweet	-	-	-	-	-	-	-	-	-	-	-
Hexanoic acid, ethyl ester	123-66-0	0.06	sweet, fruit, cellar, cucumber	-	-	-	-	-	-	-	-	-	-	-
Butanoic acid, 3-methyl-, ethyl ester	108-64-5	0.01	apple, pineapple, banana, fruit	-	-	-	-	-	-	-	-	-	-	-
Furfural	98-01-1	44.03	burnt, nutty, rancid	-	-	-	-	-	-	-	-	-	-	-
Acetic acid, 2-phenylethyl ester	103-45-7	0.91	flower, rose	4.23	3.96	3.61	2.37	2.46	1.80	1.90	1.41	<1	1.00	<1
Pyrazine, trimethyl-	14667-55-1	0.73	green pepper, coffee, toasted bread	-	-	-	-	-	-	-	-	-	-	-
Pentanoic acid, ethyl ester	539-82-2	0.03	fruit, cheese	-	-	-	-	-	-	-	-	-	-	-
Butanedioic acid, diethyl ester	123-25-1	353.19	fruit, flower, pollen	-	-	-	-	-	-	-	-	-	-	-
1-Propanol	71-23-8	314.00	fruit, flower, grass	<1	<1	<1	<1	<1	<1	<1	<1	<1	<1	<1
Pyrazine, tetramethyl-	1124-11-4	80.07	sweet, fruit	<1	<1	<1	<1	<1	<1	<1	<1	<1	<1	<1
Heptanoic acid, ethyl ester	106-30-9	13.15	flower, fruit, honey, sweet	<1	<1	<1	<1	<1	<1	<1	<1	<1	<1	<1
1-Hexanol	111-27-3	5.30	grass, resin, cream	-	-	-	-	-	-	-	-	-	-	-
Nonanoic acid, ethyl ester	123-29-5	3.15	ester, honey, fruit	<1	<1	<1	<1	<1	<1	<1	<1	<1	<1	<1
2-Furanmethanol	98-00-0	2.00	caramel, toast, burnt rubber	<1	<1	<1	<1	<1	<1	<1	<1	<1	<1	<1
Benzoic acid, ethyl ester	93-89-0	1.43	rose	<1	<1	<1	<1	<1	<1	<1	<1	<1	<1	<1
Decanoic acid, ethyl ester	110-38-3	1.12	pineapple, fruit, flower	<1	<1	<1	<1	<1	<1	<1	<1	<1	<1	<1
Isobutyl acetate	110-19-0	0.92	apple, banana	<1	<1	<1	<1	<1	<1	<1	<1	<1	<1	<1
Tetradecanoic acid, ethyl ester	124-06-1	0.50	sweet, waxy	-	-	-	-	-	-	-	-	-	-	-

Table S4. Continued. OAV table for fifth rounds of characteristic compounds ("-" indicates that this compound was not detected)

Flavor components	CAS	Threshold(mg/L)	Aroma Description	13.07%vol	12.23 %vol	11.27 %vol	10.50 %vol	9.70% vol	8.87% vol	8.53% vol	7.93% vol	7.07% vol	6.50% vol
1-Butanol, 3-methyl-	123-51-3	70.00	fruit, flower, foul odor	2.21	2.65	2.08	1.99	1.84	1.68	1.62	1.50	1.34	1.23
Propanoic acid, 2-hydroxy-, ethyl ester	97-64-3	128.08	sweet, fruit, grass	9.27	9.92	7.87	6.74	7.78	5.69	6.25	5.09	6.10	4.17
Furfuryl ethyl ether	6270-56-0	40.87	caramel, toasted bread	1.31	<1	1.03	1.15	1.06	<1	<1	<1	<1	<1
1-Propanol, 2-methyl-	78-83-1	28.26	fusel oil, malt, wine	4.52	1.05	3.80	3.68	3.40	3.11	2.99	2.78	2.48	2.28
Hexadecanoic acid, ethyl ester	628-97-7	8.54	waxy, butter	-	-	-	-	-	-	-	-	-	-
1-Butanol	71-36-3	2.73	fruit	13.16	11.94	10.18	9.79	9.04	8.27	7.95	7.39	6.59	6.06
Benzeneacetic acid, ethyl ester	101-97-3	0.41	flower, fruit, banana	-	-	-	-	-	-	-	-	-	-
Benzenepropanoic acid, ethyl ester	2021-28-5	0.13	sweet, flower	-	-	-	-	-	-	-	-	-	-
1-Butanol, 3-methyl-, acetate	123-92-2	0.09	banana, sweet, fruit candy	9.80	3.18	9.01	9.16	8.46	7.74	7.44	6.92	6.17	5.67
Butanoic acid, ethyl ester	105-54-4	0.08	apple, pineapple, fruit, floral	-	-	-	-	-	-	-	-	-	-
Butanoic acid, 2-methyl-, ethyl ester	7452-79-1	0.06	pineapple, apple, sweet	-	-	-	-	-	-	-	-	-	-
Hexanoic acid, ethyl ester	123-66-0	0.06	sweet, fruit, cellar, cucumber	-	-	-	-	-	-	-	-	-	-
Butanoic acid, 3-methyl-, ethyl ester	108-64-5	0.01	apple, pineapple, banana, fruit	-	-	-	-	-	-	-	-	-	-
Furfural	98-01-1	44.03	burnt, nutty, rancid	-	-	-	-	-	-	-	-	-	-
Acetic acid, 2-phenylethyl ester	103-45-7	0.91	flower, rose	<1	<1	<1	<1	<1	<1	<1	<1	<1	<1
Pyrazine, trimethyl-	14667-55-1	0.73	green pepper, coffee, toasted bread	-	-	-	-	-	-	-	-	-	-
Pentanoic acid, ethyl ester	539-82-2	0.03	fruit, cheese	-	-	-	-	-	-	-	-	-	-
Butanedioic acid, diethyl ester	123-25-1	353.19	fruit, flower, pollen	-	-	-	-	-	-	-	-	-	-
1-Propanol	71-23-8	314.00	fruit, flower, grass	<1	<1	<1	<1	<1	<1	<1	<1	<1	<1
Pyrazine, tetramethyl-	1124-11-4	80.07	sweet, fruit	<1	<1	<1	<1	<1	<1	<1	<1	<1	<1
Heptanoic acid, ethyl ester	106-30-9	13.15	flower, fruit, honey, sweet	<1	<1	<1	<1	<1	<1	<1	<1	<1	<1
1-Hexanol	111-27-3	5.30	grass, resin, cream	-	-	-	-	-	-	-	-	-	-
Nonanoic acid, ethyl ester	123-29-5	3.15	ester, honey, fruit	<1	<1	<1	<1	<1	<1	<1	<1	<1	<1
2-Furanmethanol	98-00-0	2.00	caramel, toast, burnt rubber	<1	<1	<1	<1	<1	<1	<1	<1	<1	<1
Benzoic acid, ethyl ester	93-89-0	1.43	rose	<1	<1	<1	<1	<1	<1	<1	<1	<1	<1
Decanoic acid, ethyl ester	110-38-3	1.12	pineapple, fruit, flower	<1	<1	<1	<1	<1	<1	<1	<1	<1	<1
Isobutyl acetate	110-19-0	0.92	apple, banana	<1	<1	<1	<1	<1	<1	<1	<1	<1	<1
Tetradecanoic acid, ethyl ester	124-06-1	0.50	sweet, waxy	-	-	-	-	-	-	-	-	-	-

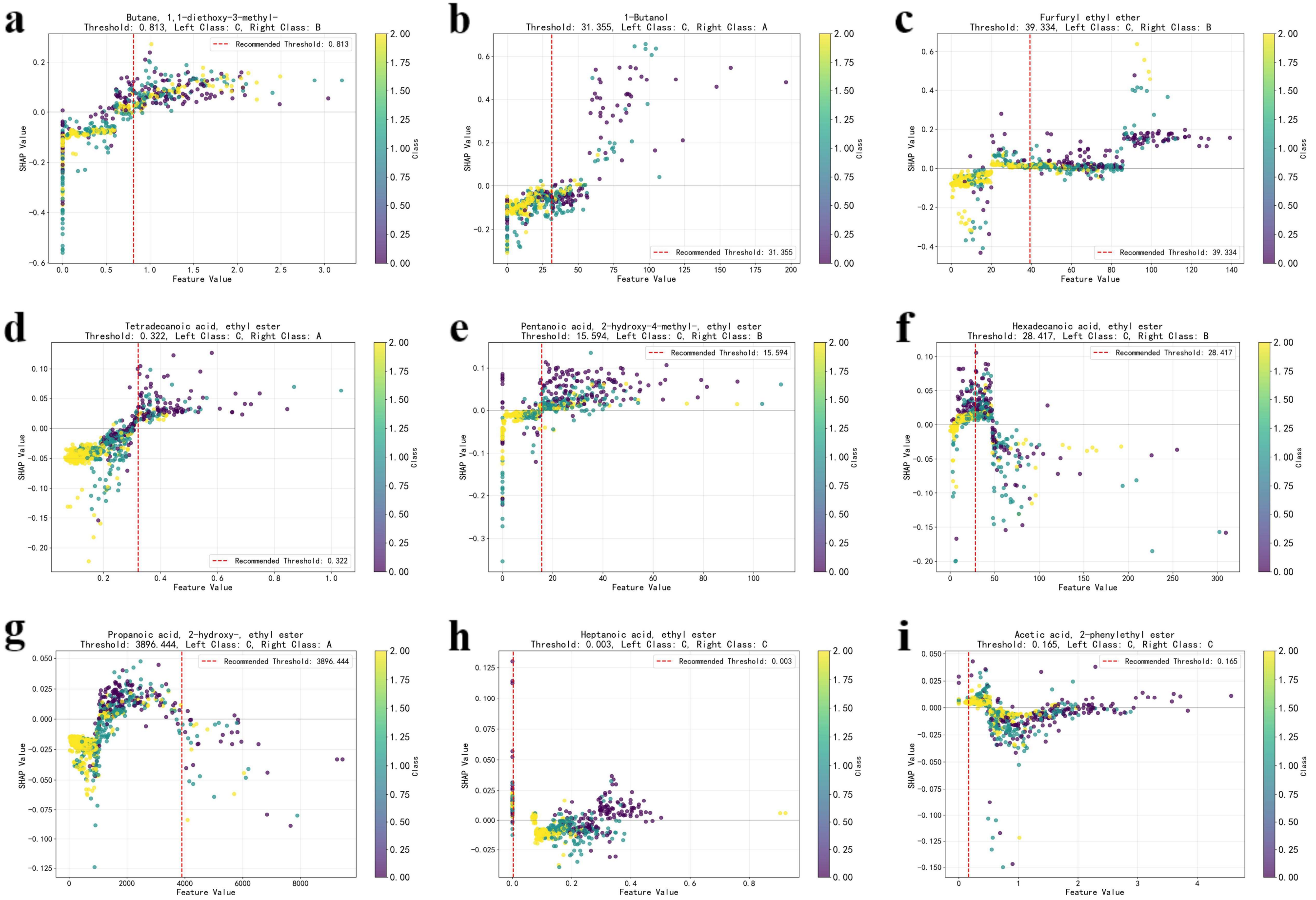

Figure S1. Characteristic compounds dependency analysis based on SHAP analysis: (a) Butane, 1,1-diethoxy-3-methyl-; (b) 1-Butanol; (c) Furfuryl ethyl ether; (d) Tetradecanoic acid, ethyl ester; (e) Pentanoic acid, 2-hydroxy-4-methyl-, ethyl ester; (f) Hexadecanoic acid, ethyl ester; (g) Propanoic acid, 2-hydroxy-, ethyl ester; (h) Heptanoic acid, ethyl ester; (i) Acetic acid, 2-phenylethyl ester.
